# Supplementary material for: Berry curvature contributions of kagome-lattice fragments in amorphous Fe–Sn thin films
Source: Nat Commun. 2023 Jun 13;14:3399. doi: 10.1038/s41467-023-39112-1 (PMC10264439; doi:10.1038/s41467-023-39112-1)
Supplement: Supplementary file 1 — Supplementary information [file 41467_2023_39112_MOESM1_ESM.pdf]

Supplementary Information

Berry curvature contributions of kagome-lattice fragments in amorphous Fe–Sn thin films

Kohei Fujiwara[1*†], Yasuyuki Kato[2†], Hitoshi Abe[3,4,5], Shun Noguchi[1], Junichi Shiogai[1,6], Yasuhiro Niwa[3,4], Hiroshi Kumigashira[3,7] Yukitoshi Motome[2], and Atsushi Tsukazaki[1,8]

[1]*Institute for Materials Research, Tohoku University, Sendai 980-8577, Japan*

[2]*Department of Applied Physics, University of Tokyo, Tokyo 113-8656, Japan*

[3]*Institute of Materials Structure Science, High Energy Accelerator Research Organization (KEK), Tsukuba 305-0801, Japan*

[4]*Department of Materials Structure Science, SOKENDAI (Graduate University of Advanced Studies), Tsukuba 305-0801, Japan*

[5]*Graduate School of Science and Engineering, Ibaraki University, Mito 310-8512, Japan*

[6]*Present address: Department of Physics, Osaka University, Toyonaka 560-0043, Japan*

[7]*Institute of Multidisciplinary Research for Advanced Materials, Tohoku University, Sendai 980-8577, Japan*

[8]*Center for Science and Innovation in Spintronics (CSIS), Core Research Cluster, Tohoku University, Sendai 980-8577, Japan*

[*]Author to whom correspondence should be addressed: kohei.fujiwara@tohoku.ac.jp

[†]These authors contributed equally to this work.

P.4. Supplementary Figure 1. Out-of-plane XRD patterns of the Fe–Sn films grown at the substrate temperature T_g = 400 °C.

P.6. Supplementary Figure 2. Scaling plots.

P.7. Supplementary Figure 3. Out-of-plane XRD patterns and x-ray reflectivity data of the Fe_xSn_{1-x} amo-films on glass.

P.8. Supplementary Figure 4. Magnetic field μ_0H dependences of magnetization M measured in an out-of-plane μ_0H and an in-plane μ_0H at T = 300 K for the $Fe_{0.74}Sn_{0.26}$ amo-film on glass.

P.9. Supplementary Figure 5. Electrical conductivity σ_{xx}, anomalous Hall conductivity σ_{AHE}, and Seebeck coefficient S_{xx} for the Fe_xSn_{1-x} amo-films and the $Fe_{0.75}Sn_{0.25}$ poly-film.

P.10. Supplementary Figure 6. The Fe K-edge EXAFS oscillations.

P.11. Supplementary Figure 7. Fourier-transformed Fe K-edge EXAFS spectra.

P.12. Supplementary Table 1. EXAFS fitting parameters.

P.13. Supplementary References

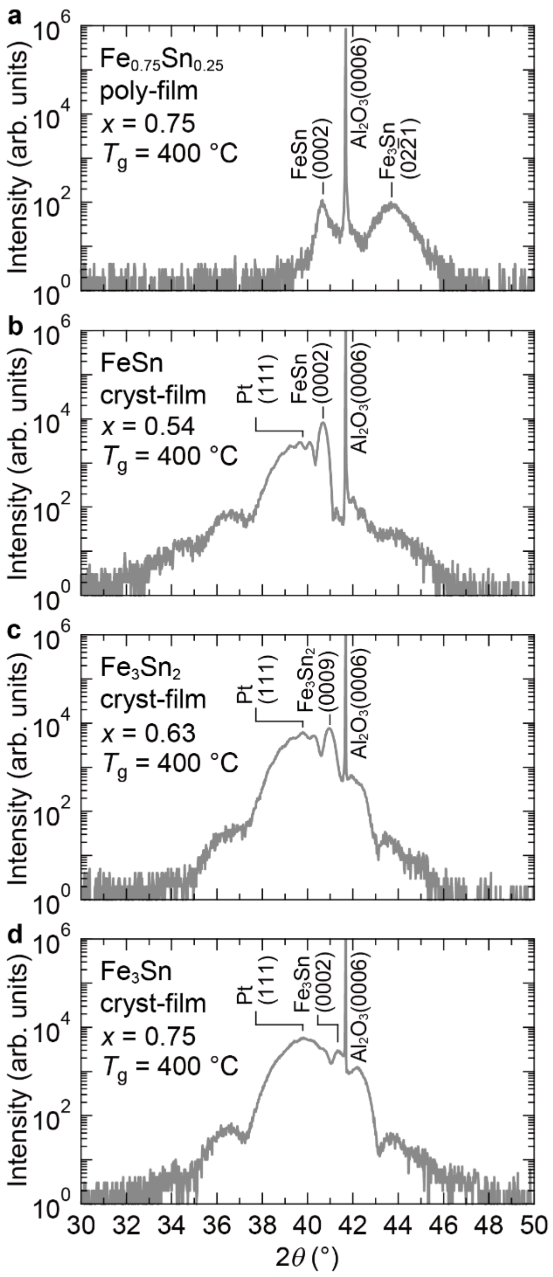

Supplementary Figure 1. Out-of-plane XRD patterns of the Fe–Sn films grown at the substrate temperature T_g = 400 °C. a $Fe_{0.75}Sn_{0.25}$ poly-film on Al_2O_3(0001). The lower-2θ and higher-2θ peaks from the film can be assigned to FeSn(0002) (JCPDS PDF No. 01-071-8400) and $Fe_3Sn(02\bar{2}1)$ (No. 01-074-5857), respectively. The *c*-axis oriented kagome-lattice Fe-Sn cryst-films on Pt/Al_2O_3(0001): **b** FeSn (x = 0.54), **c** Fe_3Sn_2 (x = 0.63), and **d** Fe_3Sn (x = 0.75). The observed diffraction angles of 2θ = 40.7° in b, 2θ = 41.0° in c, and 2θ = 41.4° in d are consistent with the reported values of FeSn(0002), Fe_3Sn_2(0009), and Fe_3Sn(0002) (Nos. 01-071-8400, 01-071-0016, 01-074-5857, respectively). The samples shown in b, c, and d were

used as the crystalline film references in the EXAFS measurements (Fig. 4b and Supplementary Figs. 6 and 7).

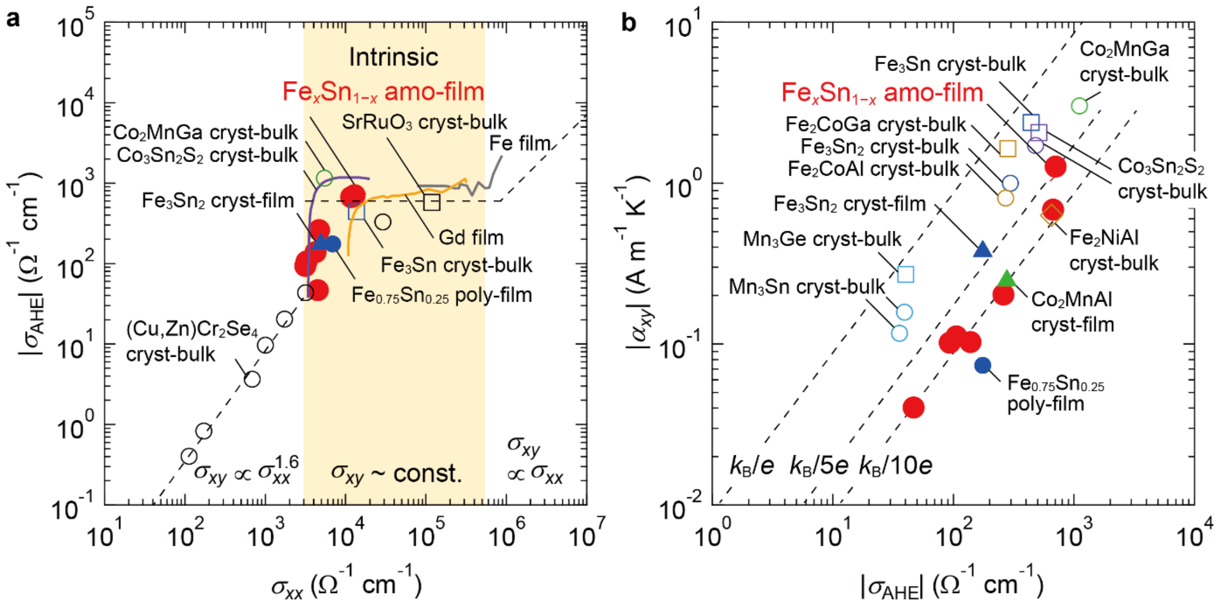

Supplementary Figure 2. Scaling plots. a Anomalous Hall conductivity σ_{AHE} versus electrical conductivity σ_{xx} plot: $Co_3Sn_2S_2$ cryst-bulk[1], Fe_3Sn_2 cryst-film[2], Fe_3Sn cryst-bulk[3], Co_2MnGa cryst-bulk[4], $SrRuO_3$ cryst-bulk, $(Cu,Zn)Cr_2Se_4$ cryst-bulk, Fe film, and Gd film[5] (see Supplementary References). The σ_{xx} of Fe_xSn_{1-x} amo-films is within the intrinsic region (mid-$10^3\ \Omega^{-1}\mathrm{cm}^{-1} \leq \sigma_{xx} \leq$ mid-$10^5\ \Omega^{-1}\mathrm{cm}^{-1}$). The σ_{AHE} is as large as those reported for topological ferromagnet crystals (kagome-lattice $Co_3Sn_2S_2$, Fe_3Sn_2, and Fe_3Sn, Heuser-type Co_2MnGa, and $SrRuO_3$). The dashed lines represent the σ_{xx} dependence of σ_{AHE} in each region. **b** Anomalous Nernst conductivity α_{xy} versus σ_{AHE} plot: $Co_3Sn_2S_2$ cryst-bulk[6,7], Fe_3Sn_2 cryst-bulk[8], Fe_3Sn_2 cryst-film[2], Fe_3Sn cryst-bulk[3], Co_2MnGa cryst-bulk[4], Co_2MnAl cryst-film[9], Fe_2CoGa, Fe_2CoAl, and Fe_2NiAl cryst-bulks[10], Mn_3Ge[11] cryst-bulk, and Mn_3Sn cryst-bulk[12]. Mn_3Ge and Mn_3Sn are antiferromagnets. The dashed lines from left to right represent the ratio of k_B/e, $k_B/5e$, and $k_B/10e$, respectively (k_B is the Boltzmann constant and e is the elementary charge).

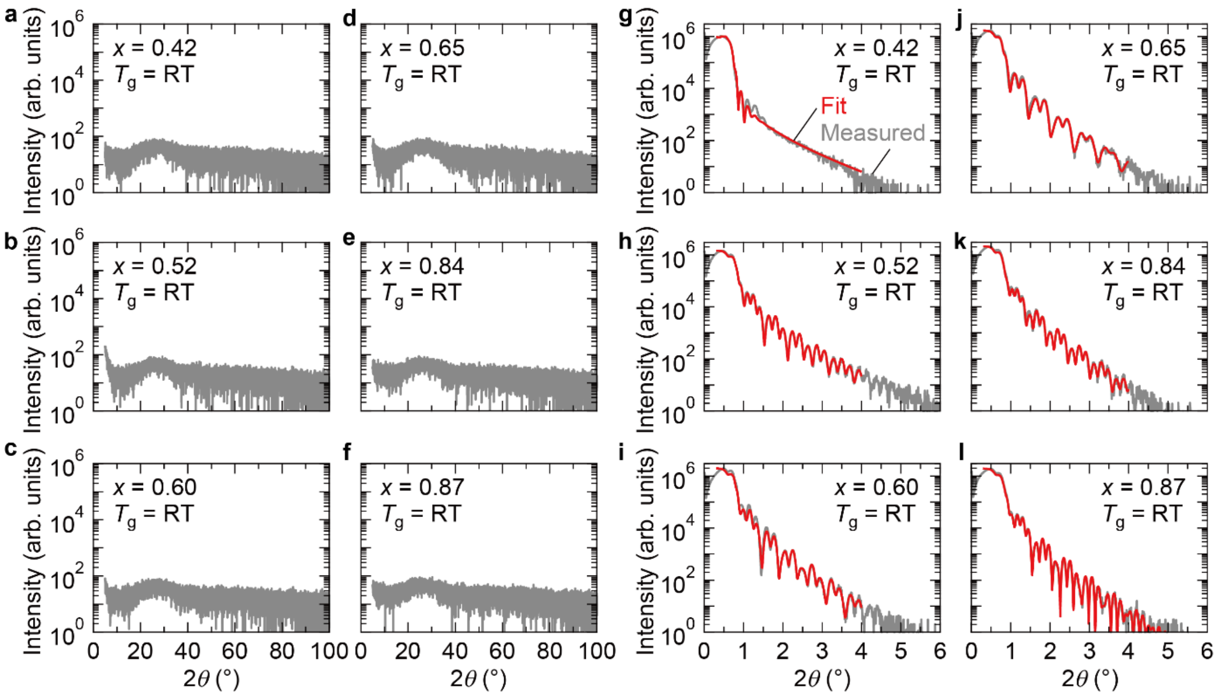

Supplementary Figure 3. Out-of-plane XRD patterns and x-ray reflectivity data of the Fe_xSn_{1-x} amo-films on glass. The substrate temperature T_g was set at room temperature (RT) for these samples. XRD: **a** $x = 0.42$, **b** $x = 0.52$, **c** $x = 0.60$, **d** $x = 0.65$, **e** $x = 0.84$, and **f** $x = 0.87$. No diffraction peaks are seen for all the samples. The broad peak around 25° comes from the glass substrate. x-ray reflectivity: **g** $x = 0.42$, **h** $x = 0.52$, **i** $x = 0.60$, **j** $x = 0.65$, **k** $x = 0.84$, and **l** $x = 0.87$. The gray and red curves in g–l are the measured data and the fitting result, respectively.

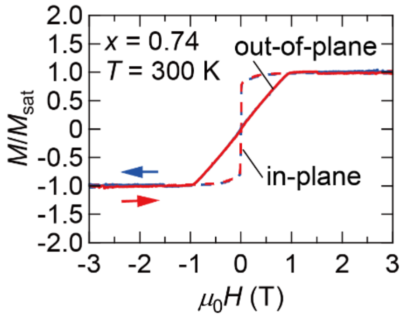

Supplementary Figure 4. Magnetic field $\mu_0 H$ dependences of magnetization M measured in an out-of-plane $\mu_0 H$ (solid blue and red curves) and an in-plane $\mu_0 H$ (dashed blue and red curves) at T = 300 K for the $Fe_{0.74}Sn_{0.26}$ amo-film on glass. The M was normalized by the saturated magnetization M_{sat} averaged over $\mu_0 H$ = 2.5–3.0 T. The blue and red curves correspond to the field-decreasing and -increasing scans.

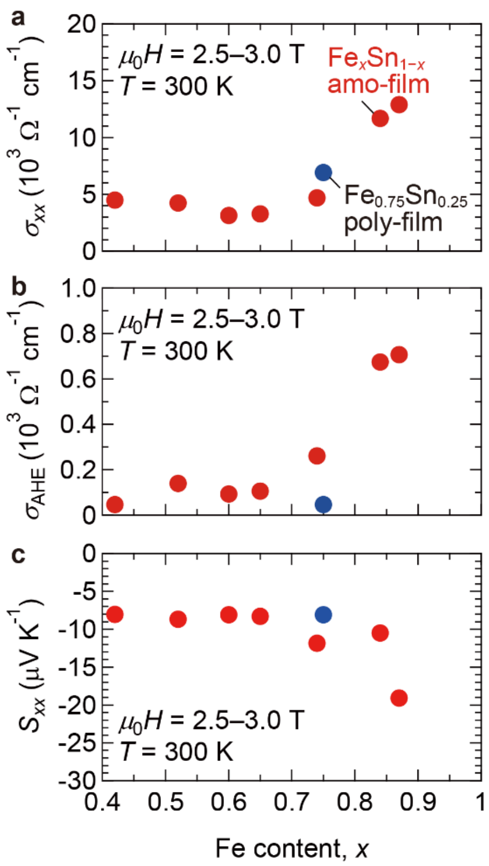

Supplementary Figure 5. a Electrical conductivity σ_{xx}, b Anomalous Hall conductivity σ_{AHE}, and c Seebeck coefficient S_{xx} for the Fe_xSn_{1-x} amo-films (shown by the closed red circles) and the $Fe_{0.75}Sn_{0.25}$ poly-film (the closed blue circles). These data are averaged for μ_0H = 2.5–3.0 T in the saturated state. These data and S_{ANE} shown in Fig. 3f are used for the calculation of α_{xy} (Fig. 3e). The error bars for these data, the standard deviations associated with the averaging, are smaller than the symbol size.

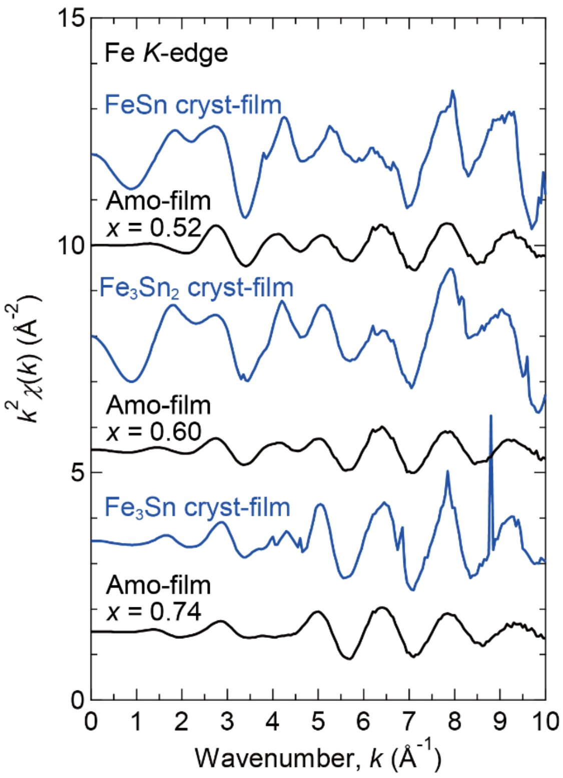

Supplementary Figure 6. The Fe *K*-edge EXAFS oscillations. The k^2-weighted EXAFS signals $\chi(k)$ of the FeSn, Fe_3Sn_2, Fe_3Sn cryst-filmst on Pt/Al_2O_3(0001) (the blue curves) and the Fe_xSn_{1-x} amo-films with x = 0.52, 0.60, and 0.74 (the black curves) are shown. The spike-like structures seen in the data of the cryst-films probably come from the diffractions of the crystalline Pt(111) buffer layer. The amo-films show similar oscillation patterns to those of the counterpart cryst-films.

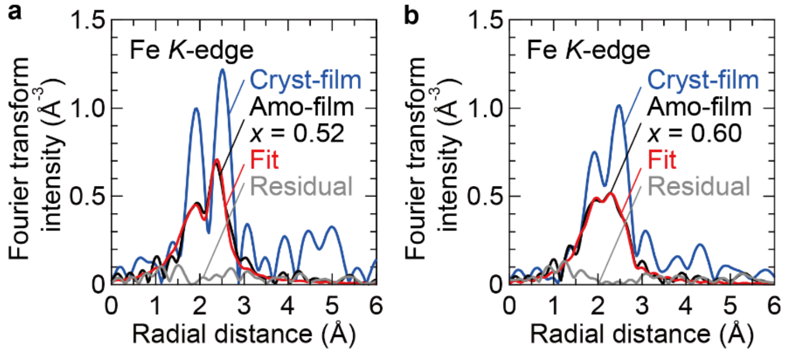

Supplementary Figure 7. Fourier-transformed Fe *K*-edge EXAFS spectra. a Amo-film with x = 0.52 (the black curve) and c-axis oriented FeSn cryst-film (the blue curve). **b** Amo-film x = 0.60 (the black curve) and c-axis oriented Fe_3Sn_2 cryst-film (the blue curve). The red and gray curves show the fitting results of the amorphous data and the residual signals, respectively.

Supplementary Table 1. EXAFS fitting parameters. The amplitude reduction factor S_0^2, the distance d and Debye–Waller factor σ^2 for the nearest neighboring Fe–Fe and Fe–Sn bonds, and normalized coordination number C.N. are shown.

Sample	S_0^2 (*)	Nearest neighboring Fe–Fe		Nearest neighboring Fe–Sn		Normalized C.N.
		d (Å)	σ^2 (Å^2)	d (Å)	σ^2 (Å^2)	
Amo-film x = 0.52	0.752	2.55	0.0127	2.55	0.0331	0.903
Amo-film x = 0.60	0.752	2.51	0.00813	2.62	0.0194	0.803
Amo-film x = 0.74	0.752	2.43	0.00567	2.70	0.0195	0.556

*The S_0^2 value determined by measuring a standard 4-µm-thick Fe bulk foil sample was fixed for these fittings.

Supplementary References

1. Liu, E. *et al.* Giant anomalous Hall effect in a ferromagnetic kagome-lattice semimetal. *Nat. Phys.* **14**, 1125–1131 (2018).

2. Khadka, D. *et al.* Anomalous Hall and Nernst effects in epitaxial films of topological kagome magnet Fe_3Sn_2. *Phys. Rev. Mater.* **4**, 084203 (2020).

3. Chen, T. *et al.* Large anomalous Nernst effect and nodal plane in an iron-based kagome ferromagnet. *Sci. Adv.* **8**, eabk1480 (2022).

4. Sakai, A. *et al.* Giant anomalous Nernst effect and quantum-critical scaling in a ferromagnetic semimetal. *Nat. Phys.* **14**, 1119–1124 (2018).

5. Miyasato, T. *et al.* Crossover behavior of the anomalous hall effect and anomalous nernst effect in itinerant ferromagnets. *Phys. Rev. Lett.* **99**, 086602 (2007).

6. Guin, S. N. *et al.* Zero-Field Nernst Effect in a Ferromagnetic Kagome-Lattice Weyl-Semimetal $Co_3Sn_2S_2$. *Adv. Mater.* **31**, 1806622 (2019).

7. Ding, L. *et al.* Intrinsic Anomalous Nernst Effect Amplified by Disorder in a Half-Metallic Semimetal. *Phys. Rev. X* **91**, 041061 (2019).

8. Zhang, H., Xu, C. Q. & Ke, X. Topological Nernst effect, anomalous Nernst effect, and anomalous thermal Hall effect in the Dirac semimetal Fe_3Sn_2. *Phys. Rev. B* **103**, L201101 (2021).

9. Sakuraba, Y., Hyodo, K., Sakuma, A. & Mitani, S. Giant anomalous Nernst effect in the $Co_2MnAl_{1-x}Si_x$ Heusler alloy induced by Fermi level tuning and atomic ordering. *Phys Rev B* **101**, (2020).

10. Mende, F. *et al.* Large Anomalous Hall and Nernst Effects in High Curie-Temperature Iron-Based Heusler Compounds. *Adv. Sci.* **8**, 2100782 (2021).

11. Xu, L. *et al.* Finite-temperature violation of the anomalous transverse Wiedemann-Franz law. *Sci. Adv.* **6**, eaaz3522 (2020).

12. Ikhlas, M. *et al.* Large anomalous Nernst effect at room temperature in a chiral antiferromagnet. *Nat. Phys.* **13**, 1085–1090 (2017).
